# Prussian blue electrocatalyst in bioanalysis: From oxidase biosensors to peroxidase kinetics assays

**DOI:** 10.1016/j.isci.2026.117419

**Published:** 2026-10-22

**Authors:** Hucheng Chang, Jianrui Han, Yuhang Zhang, Qicong Tang, Yue Li, Yan Zhang, Ting Wei, Jiao-Jiao Du, Su Ma

**Affiliations:** 1 Department of Life Sciences, Changzhi University, Changzhi, China; 2 Department of Chemical and Process Engineering, MacDiarmid Institute for Advanced Materials and Nanotechnology, University of Canterbury, Christchurch, New Zealand; 3 State Key Laboratory of Microbial Technology, Shandong University, Qingdao, China

**Keywords:** Electrochemistry, Electrocatalyst, Prussian Blue, Nanozymes, Biosensors, Peroxidase, Oxidase

## Abstract

Prussian blue (PB) has evolved as the most advantageous electrocatalyst for preparing H_2_O_2_ biosensors due to its exceptional high sensitivity and specificity, which are vital to minimizing the interferences in complex biological samples. However, the widespread adoption of PB-based bioanalysis has been severely restricted by its inherent instability in neutral and alkaline environments, compromising the measurement accuracy and reliability. This review provides an overview of the strategies used to improve PB stability in bioanalysis, such as integration with carbon nanomaterials, polymer encapsulation (e.g., Nafion and chitosan), and transition metal ion (e.g., Ni, Co)-doping. Furthermore, we comprehensively cover the bioanalytical applications of PB: from traditional oxidase-based biosensors to emerging high-impact applications enabled by the enhanced PB stability. These includ real-time H_2_O_2_ quantification in tumors and brain tissue, electrochemical immunosensing, and kinetics assays for peroxidases and peroxygenases acting on insoluble substrates.

## Graphical abstract

**Figure:**
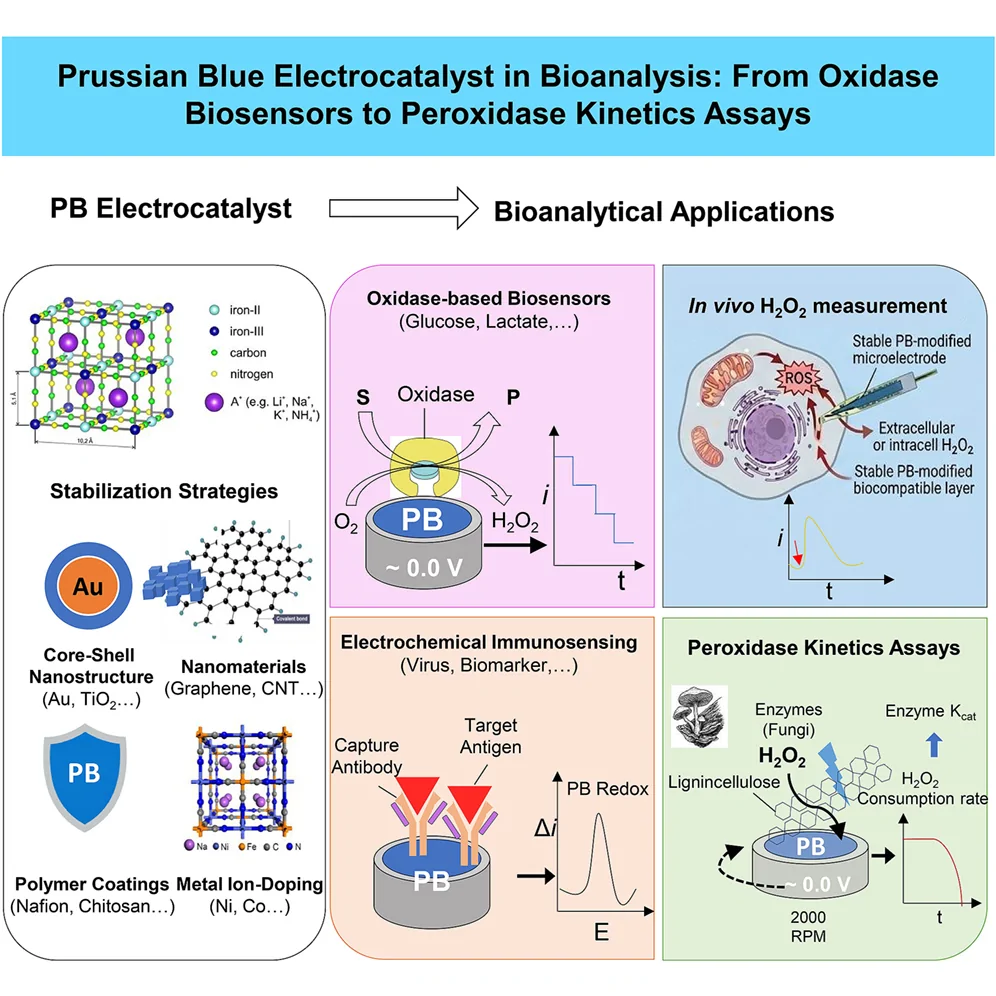

## Teaser

Biochemistry; Bioelectronics; Electrochemistry; Nanomaterials

## Introduction

Prussian blue (PB), discovered in the early 18^th^ century as the first synthetic blue pigment, is a coordination compound composed of ferric (Fe^3+^) and ferrous (Fe^2+^) ions bridged by cyanide ligands. This arrangement forms a rigid cubic lattice with a unit-cell dimension of approximately 10.2 Å .^1^ A pivotal milestone in PB research occurred in 1978, when Vernon D. Neff elucidated its electroactive properties and electrochromic behavior across both anodic and cathodic potentials.^2^ Karyakin subsequently demonstrated that PB acts as a highly specific electrocatalyst for H_2_O_2_ reduction in the presence of oxygen. This exceptional catalytic specificity allows PB to outperform traditional iron oxide or platinum-based catalysts, positioning it as a premier “artificial peroxidase” in bioanalytical chemistry.^3^^,^^4^^,^^5^^,^^6^

Despite the outstanding activity and specificity, PB exhibits intrinsic structural and electrochemical instability, particularly in alkaline or highly ionic solution,^7^^,^^8^ restraining its broader utilization. The framework’s susceptibility to partial dissolution, lattice defects, and Fe–CN bond cleavage could severely degrade its catalytic performance,^9^ and therefore compromise the accuracy and reproducibility of PB-based biosensors. Recent studies have significantly improved PB stability through metal ion-doping,^10^^,^^11^ polymer or carbon-based encapsulation,^12^^,^^13^ and core-shell nanoarchitectures.^14^ The improved material stability and catalytic durability have allowed PB to transition from traditional oxidase-based biosensors to diverse applications, for example, determination of cellular oxidative stress,^15^ high-sensitivity immunosensors,^16^ and the emerging peroxidases (and peroxygenases) kinetics assays.^17^ In this review, we critically discuss those stabilization strategies for improving PB-based electrocatalysts activity and their applications in traditional and emerging areas, which include oxidase-biosensors, cellular oxidative stress, immunosensing, peroxidase kinetics assays.

## PB structure

The microcrystalline structure of PB was first solved in 1936 using powder X-ray diffraction analysis.^18^ The cubic lattice of PB, featuring the alternative distribution of ferrous (Fe^2+^) and ferric (Fe^3+^) atoms at the corners of a 5.1 Å cube, which are linked by bidentate (–C≡N–) ligands (Figure 1). Each crystallographic unit cell comprises eight cubes and has a lattice parameter of approximately 10.2 Å, but the specific structure and chemical composition of PB vary slightly with the reactants and the precipitation rate during synthesis. The characteristic blue color of PB arises from an intervalence charge-transfer transition from the Fe^2+^ to the Fe^3+^ ions, which leads to strong absorption in the orange-red region of the visible spectrum (680–730 nm).^20^ Beyond simply visual inspection, Raman and infrared spectroscopies (IR) have been widely employed to characterize PB due to the presence of distinctive (C≡N) stretching bands and the metal-cyanide coordination environment. In addition, Fe-Mössbauer spectroscopy provides a powerful tool for resolving iron oxidation states and especially for distinguishing the reduced and oxidized forms of PB owing to the valence dynamics of iron atoms in the PB lattice during electrochemical processes. For a comprehensive discussion of PB material characterization, we recommend the chapter in the book “Prussian Blue Based Batteries”.^21^

**Figure 1:**
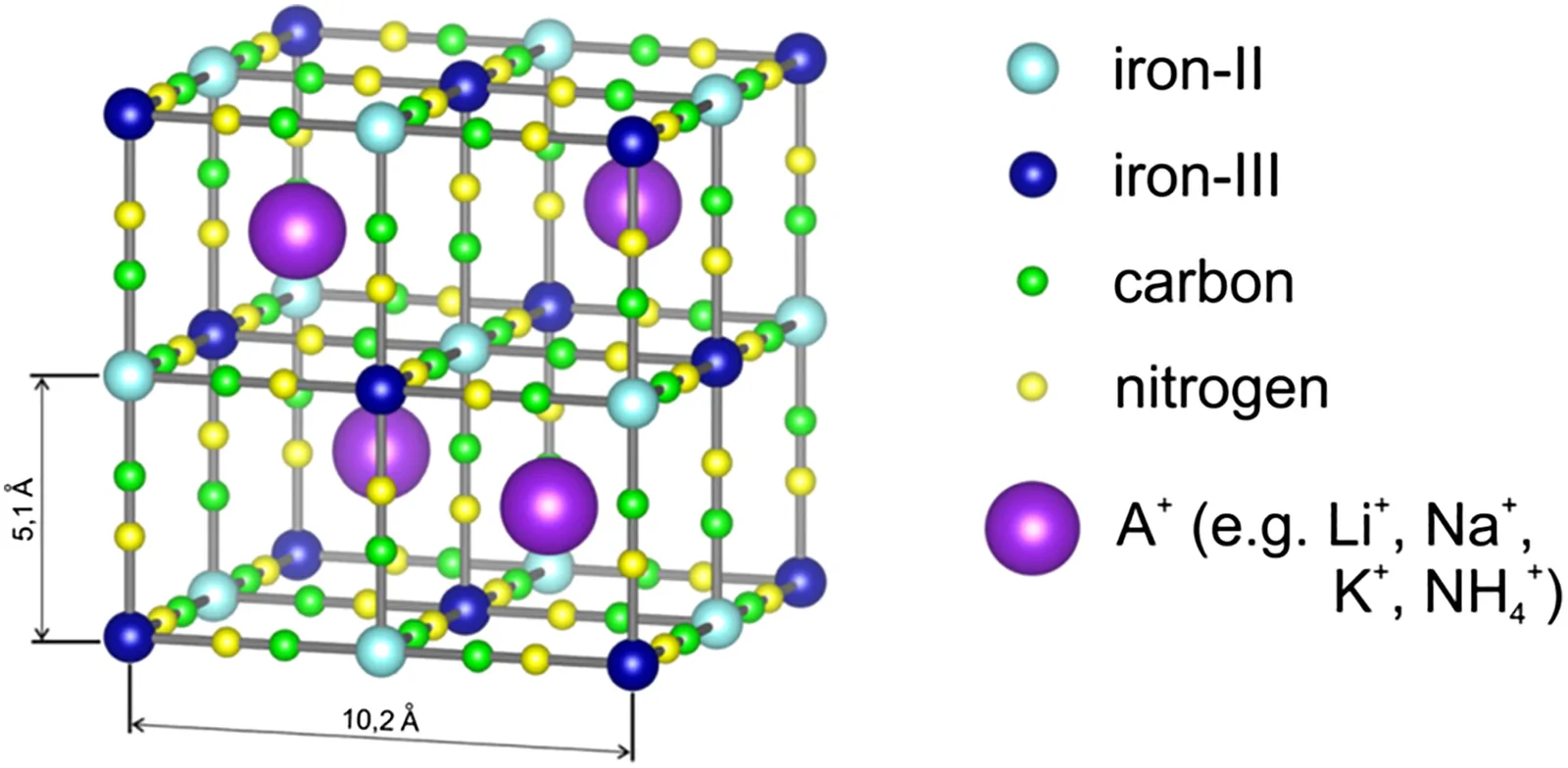
Chemical structure of PB with 4 alkali-metal ions in the middle of octants (“soluble” form), while 1 Fe^3+^ instead of 4 A^+^ is proposed for the “insoluble” form Reprinted from Kraft A.^19^ with the permission of Springer Nature.

## Electrochemistry of PB

The exceptional utility of PB in electrochemical systems stems from its unique crystal structure, which is a face-centered cubic lattice where iron ions in different oxidation states are bridged by cyanide ligands. This framework allows for highly reversible solid-state redox transitions, characterized by the insertion and extraction of alkali metal cations (e.g., K^+^, Na^+^) to maintain electroneutrality. In the presence of hydrated cations like K^+^, the PB films on electrodes undergo two main transitions: (1) oxidation of PB to Berlin green and (2) reduction of PB to Prussian white (PW).^7^

### Reduction to PW

The reduction of the high-spin Fe^III^ ions to low-spin Fe^II^ occurs at relatively low potentials (approx. 0.2 V vs. Ag|AgCl). This transition converts the blue film into a colorless, transparent state known as PW as shown in the below equation:

(Equation 1) $${KFe}^{III}{[Fe}^{II}{\left(CN\right)}_{6}]+e^{-}+K^{+}↔K_{2}{Fe}^{II}\left[{Fe}^{II}{\left(CN\right)}_{6}\right]\text{.}$$

This specific transition is the foundation for electrochemical H_2_O_2_ sensing, as it provides the electrons required for catalytic reduction (Figure 2A).

**Figure 2:**
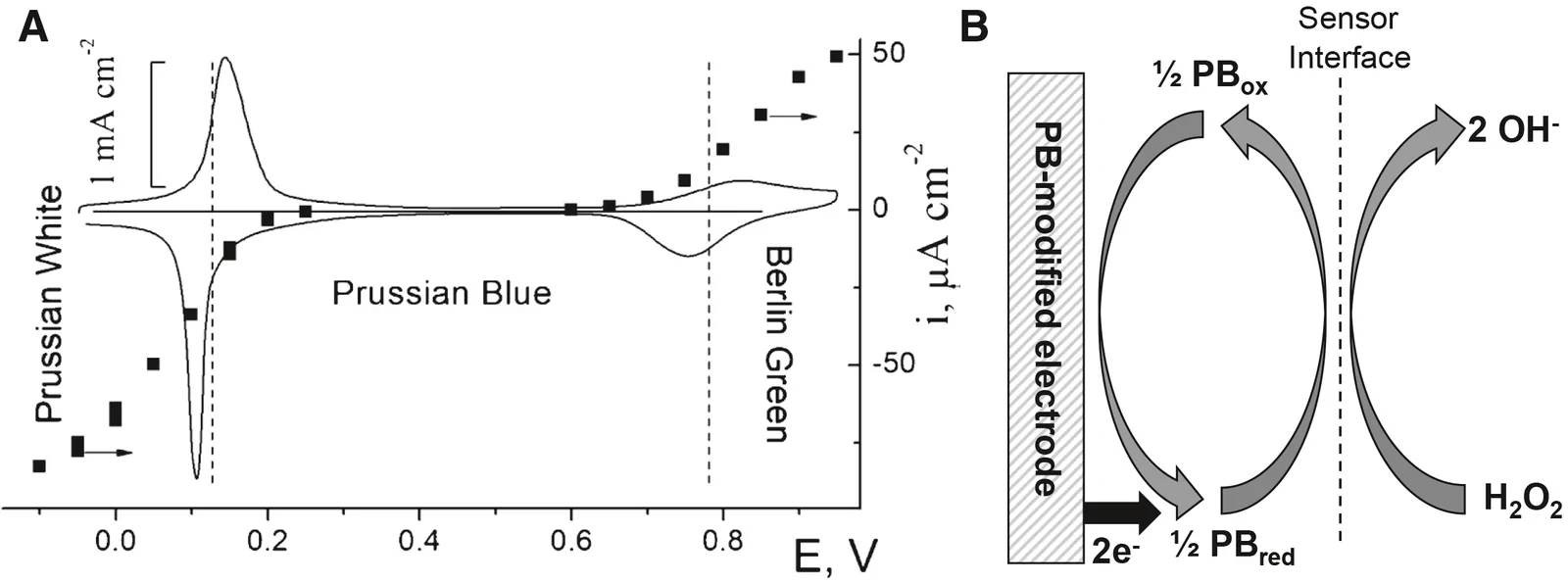
Electrochemistry of PB film and its electrocatalytic reduction of H_2_O_2_ (A) Cyclic voltammogram of PB-modified electrode at a scan rate of 40 mV s^−1^ and steady-state current (square dots) in the presence of 0.1 mM H_2_O_2_ in 1 mM HCl with 100 mM KCl. Reproduced from Komkova et al.,^4^ with the permission of Springer Nature. (B) Electrochemical reduction of H_2_O_2_ catalyzed by PB.

### Oxidation to Berlin green

At higher potentials (approx. 0.9 V vs. Ag|AgCl), the low-spin Fe centers are oxidized to Fe^III^, leading to the formation of Berlin green species:

(Equation 2) $${KFe}^{III}{[Fe}^{II}{\left(CN\right)}_{6}]↔{Fe}^{III}{\left[{Fe}^{III}{\left(CN\right)}_{6}\right]}_{2/3}\;{\left[{Fe}^{II}{\left(CN\right)}_{6}\right]}_{1/3}+e^{-}+K^{+}\text{.}$$

Across both steps, the electrochemical cycle consists of electron transfer, cation insertion or extraction within the crystal lattice, reorganization of interstitial water molecules, and subtle distortions within the cyanide-bridged network.^22^ Importantly, the quality of PB film in terms of crystallinity, thickness, defect concentration, and hydration level determines the reversibility and kinetics of these transformations.

## Electrocatalyst for H_2_O_2_ reduction

The extensively bioanalytical applications of PB are owing to its high activity toward the reduction of H_2_O_2_, which outperforms even natural peroxidases.^4^ Unlike commonly used noble metal catalysts (e.g., Pt, Au, and Ag), which exhibit nearly the same activity for the reduction of both H_2_O_2_ and O_2_, PB shows high selectivity for H_2_O_2_ reduction over oxygen reduction reaction.^23^^,^^24^ For this reason, PB is often termed “artificial peroxidase” in the contexts of bioanalytical and biomedical applications. This “H_2_O_2_ reduction” detection mode is critical for biosensing applications because it avoids the oxidation of common biological interferences like ascorbic acid, glutamate, and acetaminophen.^23^^,^^25^ During the catalysis, H_2_O_2_ reacts with the PW, oxidizing it back to PB while the H_2_O_2_ is reduced to OH^−1^. Subsequently, the electrode then provides a cathodic current to reduce the PB back to PW, completing the cycle.^9^ Therefore, the peroxidase-like catalysis of PB is supposedly driven by proper electron donors (e.g., cathodic potentials) (Figure 2B).

## PB synthesis—Chemical precipitation

PB is typically synthesized via chemical co-precipitation or electrochemical deposition using ferric salts and hexacyanoferrate complexes in an acidic medium, such as HCl. Although the fundamental reaction involves the coordination of Fe^3+^ ions with ferrocyanide or ferricyanide complexes, practical protocols vary significantly in precursor composition and synthetic conditions (e.g., pH and temperature). In precipitation processes, these factors determine the particle size, crystallinity, and hydration level of the resulting PB, which directly impact its catalytic activity and structural stability. Traditionally, ferric salt is mixed with the equimolar ferrocyanide ions at room temperature, which results in rapid nucleation and formation of PB precipitates. However, this straightforward protocol often yields poorly controlled morphologies and a broad size distribution, typically ranging from hundreds of nanometers to several microns (Figure 3).^27^^,^^28^ The solution pH is critical, as PB forms optimally at mildly acidic conditions (pH 2–4), where the hydrolysis of Fe^3+^ is suppressed while the formation of Fe–CN–Fe linkages is promoted.^29^ Furthermore, elevated temperatures and pressures can enhance ion diffusion, facilitating the growth of well-ordered crystals. For detailed discussions on synthetic pathways involving varied thermal treatments or the substitution of transition metals (e.g., Co, Ni, Mn, and Cu) to form PB analogs, we refer readers to the recent reviews.^30^^,^^31^

**Figure 3:**
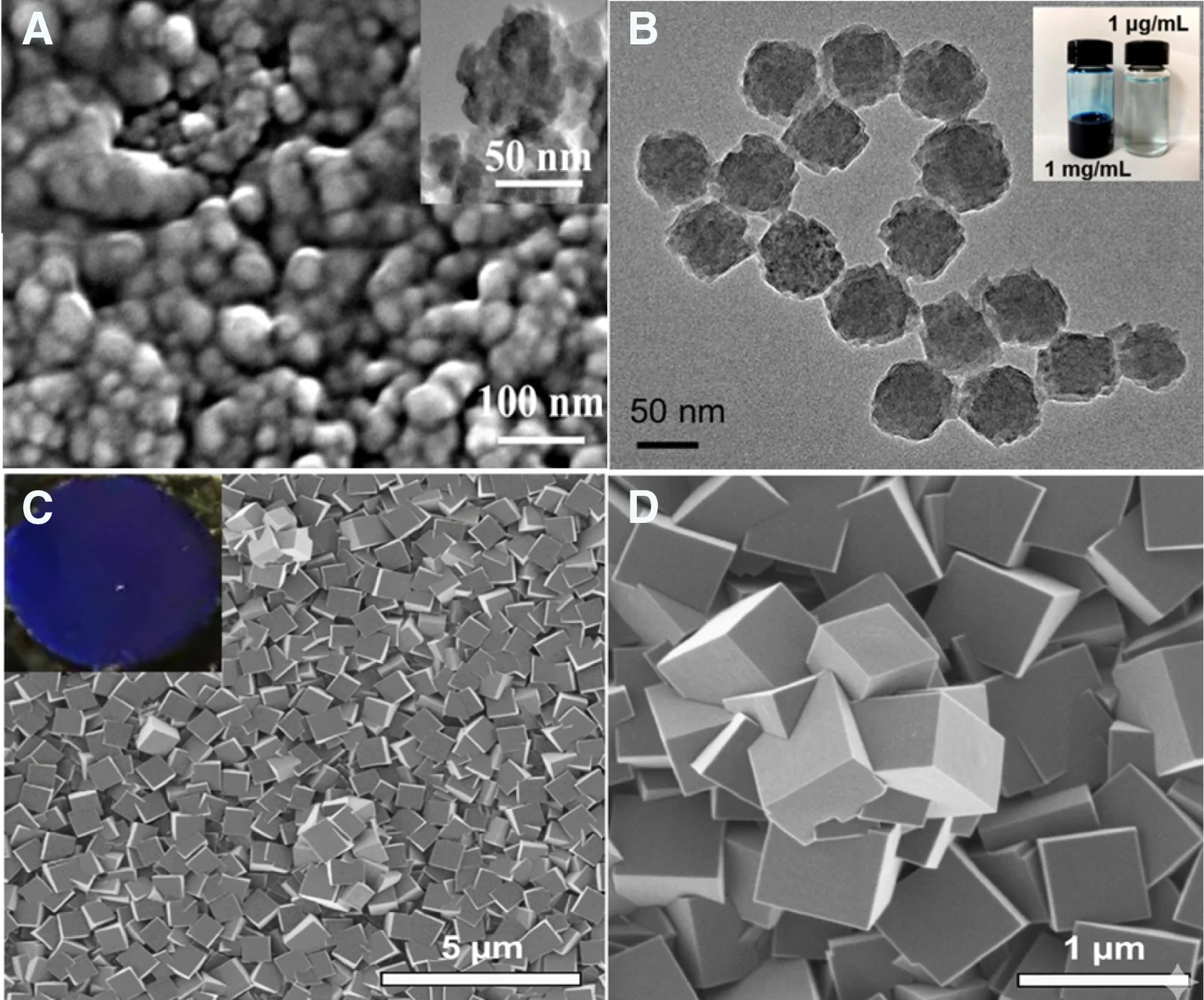
Microscopy of PB nanoparticles in solution and PB film on electrode (A) Scanning electron microscopy (SEM) and transmission electron microscopy (TEM) images of the catalytically synthesized PB nanoparticles with an average diameter of ≈40 nm. Reproduced from Komkova et al.,^3^*Journal of the American Chemical Society*, 2018. (B) TEM image of PB nanozymes. Inset: digital photograph of the PB nanozymes aqueous solution. Reproduced from Dong et al.,^26^*Analyst*, 2022. (C and D) SEM images of PB films that were electrochemically deposited at 0.3 V. Inset in (C): digital photograph of the deposited PB film on Au/Cr/Si chip electrode. Reproduced from Avila et al.^27^ with the permission of MDPI.

Recently, highly uniform PB nanozymes have attracted significant attention, due to their exceptional peroxidase-like activity.^32^^,^^33^ Komkova et al. introduced a synthesis protocol utilizing a reductant, such as H_2_O_2_ or organic molecules, to produce more regular nanostructures by reducing ferricyanide-ferric ion mixtures^3^ (Figure 3A). In this protocol, reduction of ferricyanide-ferric ions mixture could produce more regular nanostructures. This approach significantly improves both catalytic activity and material stability for electrochemical applications, yielding PB nanoparticles with catalytic constants up to four orders of magnitude higher than natural peroxidases for H_2_O_2_ reduction reaction. Additionally, auxiliary ligands, such as polyvinylpyrrolidone (PVP),^34^ polyvinyl alcohol (PVA),^35^ polyethylene glycol (PEG),^36^ or citrate^37^ serve as capping agents to inhibit the aggregation of nanoparticles, yielding stabilized nanoparticles with diameters as small as 50 nm, which are commonly used for preparing PB-based nanozymes (Figure 3B).

## PB synthesis—Electrochemical deposition

Electrochemical deposition is the preferred method for preparing PB-modified electrodes in biosensing applications. This approach allows for the production of adherent, uniform films with precisely controllable thickness using galvanostatic, potentiostatic, or cyclic voltammetry (CV) techniques (Figures 3C and 3D). During potentiostatic deposition, a constant potential, typically ranging from +0.25 to +0.4 V (vs. Ag|AgCl). drives the redox reactions of the iron cyanide at the electrode surface to ensure optimal crystallinity and activity.^38^^,^^39^ The other parameters, such as deposition time, electrode conductivity, and the precursors’ concentrations, also impact the film thickness and the resulting catalytic activity. Alternatively, galvanostatic deposition parameters vary according to electrode surface area and the concentration of ferricyanide-ferric precursor.^40^ CV-based deposition is achieved by repeated potential cycling to switch between the redox states of iron-cyanide complexes, promoting layer-by-layer film formation (Figure 4A). Because the nucleation of PB occurs preferentially at certain potential ranges, CV-deposited films often exhibit superior crystallinity and optimal incorporation of water molecules, which are essential for ion mobility within the PB framework.

**Figure 4:**
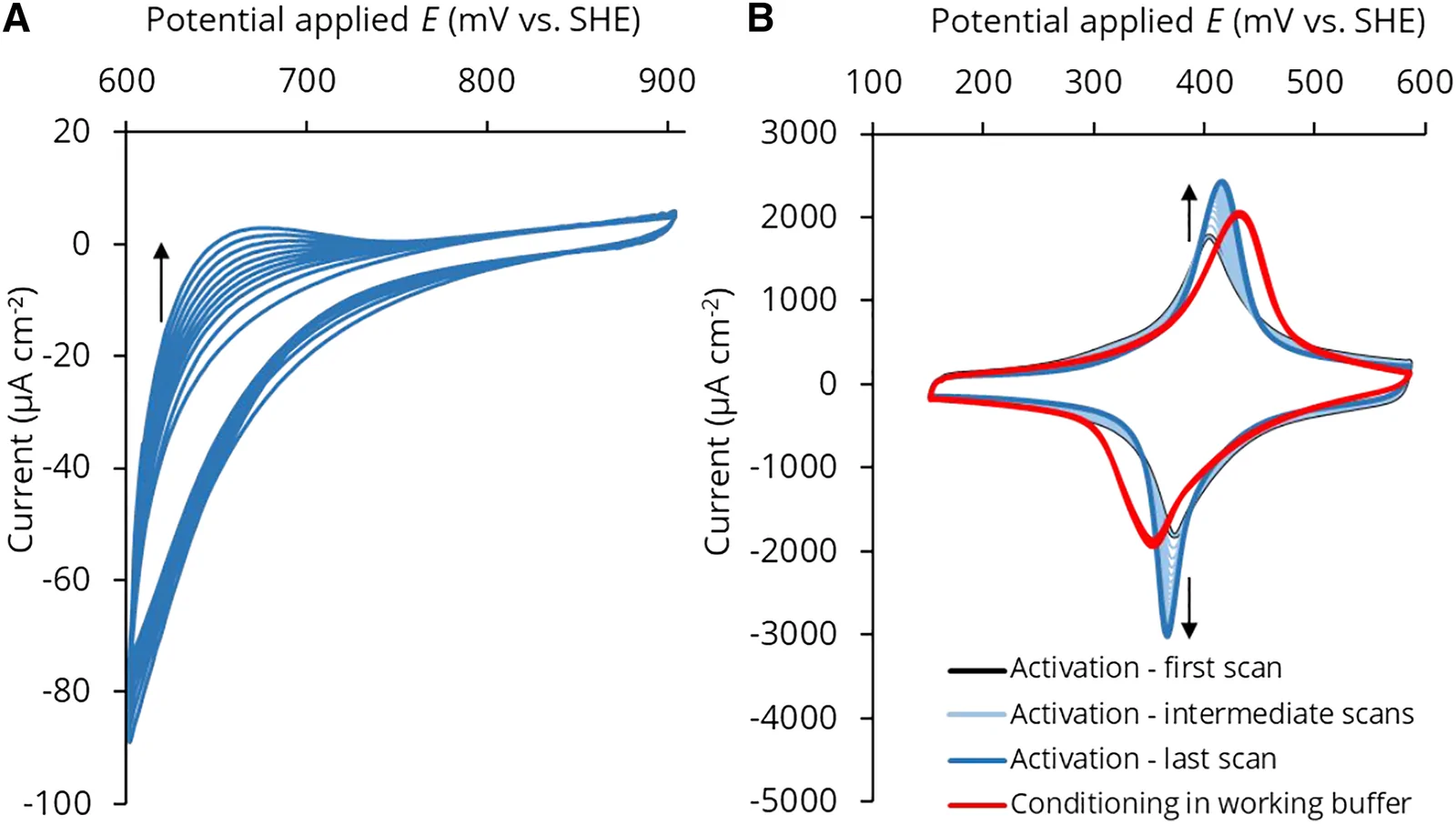
Preparing PB film with cyclic voltammetry (A) The PB film was deposited on a gold electrode by CV scanning at 20 mV s^−1^ in a solution containing 1 mM FeCl_3_, 1 mM potassium ferricyanide (K_3_[Fe(CN)_6_]), 100 mM KCl, and 100 mM HCl. (B) Activation of the deposited PB electrocatalysts by cycling at a scan rate of 50 mV s^−1^ in the supporting electrolyte. Reprinted from Schwaiger et al.,^17^*ACS Catalysis*, 2024.

Regardless of the deposition technique, PB films on electrodes often require an activation procedure—typically potential cycling (e.g., −0.05 − +0.35 V vs. Ag|AgCl)—in the supporting electrolyte. Successful activation is indicated by the appearance of sharp, characteristic PB redox peaks in the cyclic voltammograms (Figure 4B). Electrodeposition is particularly advantageous for oxidase-based platforms as it allows for direct growth on substrates like Au, Pt, glassy carbon, Indium Tin Oxide (ITO), or carbon nanotubes (CNTs) without the need for binders that might impair conductivity or PB lattice integrity. The synthesis of PB encompasses a diverse landscape of methodologies. While chemical co-precipitation remains the most scalable approach, electrochemical deposition offers unparalleled control for biosensor integration in the subsequent applications. As research shifts toward PB nanozymes and functional analogs, continued innovation in synthesis will be vital for optimizing stability and application-specific performance.^41^

## Mechanisms of PB instability

PB exhibits distinct instability phenomena during electrochemical reactions in alkaline solution, especially during repeated charge-discharge cycles.^42^^,^^43^ The instability of PB originates from both structural degradation and the leaching of active components into the electrolyte. The chemical vulnerability of the cyano-bridged framework to nucleophilic attack is strongly dependent on pH. In acidic media (pH < 4), PB exhibits remarkable stability, but the lattice undergoes progressive degradation at the physiological pH (7.4), which is typically preferable for oxidase-based biosensing. This process is predominantly caused by hydroxyl ions (OH^−^), which attack the ferric (Fe^3+^) sites within the frameworks of the PB lattice. While the alkaline decomposition of the PB lattice is commonly summarized by the net stoichiometric relationship shown in Equation 3, it must be emphasized that this represents a highly simplified schematic of the overall reaction rather than a single-step pathway:

(Equation 3) $${Fe}_{4}{\left[Fe{\left(CN\right)}_{6}\right]}_{3}+12{OH}^{-}\to 4Fe{\left(OH\right)}_{3}+3{\left[{Fe}^{II}{\left(CN\right)}_{6}\right]}^{-4}\text{.}$$

In reality, the degradation is a multi-step interfacial process that initiates with the nucleophilic attack of hydroxyl ions (OH^−^) targeting high-spin (Fe^3+^) sites situated at the boundaries or defect zones of the crystalline framework. This attack triggers the sequential cleavage of the bridging Fe^II^‒C≡N‒Fe^III^ coordination bonds. As the structural integrity of the lattice compromises, the highly stable, low-spin low-potential hexacyanoferrate species ([Fe(CN)_6_]^4−^) are progressively liberated into the bulk solution. Concurrently, the remaining desorbed, uncoordinated Fe^3+^ ions undergo rapid localized hydrolysis and subsequent precipitation, forming amorphous ferric hydroxide. This phase transition ultimately leads to the complete macroscopic dissolution and loss of electrocatalytic activity of the PB modifier layer under neutral-to-alkaline conditions.

Furthermore, stability issues are exacerbated during the electrocatalytic reduction of H_2_O_2_.^44^ Recent studies using scanning electrochemical microscopy have demonstrated that this catalysis generates local OH^−^ ions and even the highly reactive hydroxyl radicals (·OH) via Fenton-like side reactions.^45^ These locally produced species create a microenvironment of high alkalinity at the electrode, accelerating the dissolution of the PB film even when the bulk solution is buffered at an acidic pH. This “self-poisoning” effect results in a rapid decrease in sensitivity and a loss of the electroactive material over repeated sensing cycles, compromising the accuracy and reproducibility of continuous measurements.

The degradation of PB films might also occur during the coverage of an enzyme layer for biosensor fabrication. During the formation of enzyme coating, the PB film is subject to sharp change in pH leading to partial dissolution of the PB crystals. Additionally, at the bio-interface between enzymes and PB films, the crystalline integrity could be damaged by the strong interactions with biomolecules.^46^

## Strategies for improving PB stability

### Protective coatings

A widely adopted approach involves encapsulating PB within protective matrices that act as semi-permeable membranes allowing the diffusion of H_2_O_2_ and OH^−^ while preventing the leaching of [Fe(CN)_6_]^−4^. Hydrophobic or ion-exchange polymers such as Nafion, chitosan, or polypyrrole have shown significant success by forming a protective coating over PB-modified electrodes. Nafion acts as a cation-exchanger that stabilizes K^+^ ions within the PB lattice, while its hydrophobic backbone blocks the leaching of [Fe(CN)_6_]^4-^ ions.^47^^,^^48^ For example, Nafion coating significantly improved the durability of PB films on gold electrodes, allowing for accurate H_2_O_2_ measurement in lignocellulose slurries over 12 h. This technique has enabled the real-time determination of the kinetics of lignin-degrading peroxidase^49^ and cellulose-degrading peroxygenase^17^ for their insoluble lignocellulosic substrates. Given that Nafion is also an excellent matrix for enzyme immobilization, Nafion-dispersed oxidase coatings are frequently employed to cover PB-modified electrode for preparing biosensors.^50^^,^^51^

Similarly, chitosan forms insoluble films in the neutral and alkaline environments, and its mechanical flexibility improves adhesion to diverse electrode surface. Additionally, its porous microstructure enhances the loading capacity for enzymes.^52^^,^^53^^,^^54^ Barber et al. developed a PB-chitosan network by one-step electrodeposition on graphene surface for the detection of H_2_O_2_ in human perspiration.^55^ Such architectures have been successfully extended to oxidase-based biosensors for detecting metabolites in complex biological fluids where pH fluctuations might otherwise compromise the PB lattice. For example, Baretta et al. employed a carboxymethyl cellulose matrix for integrating PB and glucose oxidase (GOx) or alcohol oxidase, and the resulting biosensors were capable of rapid and sensitive detection of glucose and ethanol in serum.^56^ Lee et al. employed the one-step copolymerization method to synthesize PB-polydopamine-polypyrrole, showing both good stability and apparent selectivity to H_2_O_2_ than other biological molecules. Because the used protocol involves simultaneous PB nucleation and heterodimeric formation, the original PB crystal structure was distorted, resulting in a lower catalytic activity.^57^

### PB-based nanocomposites

In addition to polymer coatings, the integration of PB with different nanomaterials to form hybrid PB-nanocomposites has emerged as a cornerstone strategy for overcoming its inherent instability while simultaneously enhancing its electrocatalytic performance. Among them, CNTs and graphene (chemically reduced graphene oxide) are perhaps the most extensively investigated.^12^^,^^15^^,^^58^^,^^59^^,^^60^^,^^61^ These carbon nanomaterials provide a high-surface-area, highly conductive 3D framework that serves as a “mechanical anchor” for PB particles. Zou et al. developed a new route to deposit polyaniline-PB hydride composites on multi-walled CNTs, which showed remarkably high sensitivity and also provided a robust platform for the fabrication of the glucose biosensor with a wide linear range.^62^

Beyond carbonaceous supports, the incorporation of metallic nanoparticles and metal oxides, such as gold nanoparticles (Au NPs) and titanium dioxide (TiO_2_), offers unique structural and functional advantages for PB stabilization (Table 1). Au NPs are particularly valued for their ability to facilitate electron tunneling between the PB redox centers and the underlying electrode surface, significantly boosting the sensitivity of the resulting biosensors.^78^^,^^79^^,^^80^ Additionally, the biocompatibility of Au NPs provides an ideal platform for the subsequent immobilization of enzymes through well-established thiol chemistry, ensuring the structural integrity of the entire bio-interface.^81^ Similarly, metal oxides like TiO_2_ have been utilized to construct core-shell or layered architectures where the oxide layer serves as a protective inorganic shield. This TiO_2_ matrix can effectively mitigate the susceptibility of the Fe–CN–Fe bonds to hydroxyl ion attack, thereby extending the operational lifespan of PB-based electrodes in neutral or slightly alkaline environments.^65^ However, many studies have combined several kinds of nanomaterials in the PB-based hybrids, where the synergy effect was not clearly proved and the final results were mostly unsatisfied. Therefore, we will not cover those works in this review.

**Table 1:** PB-based glucose biosensors

PB-based material (synthesis)	LOD (μM)	Working pH	Sensitivity (mA/M cm^2^)	Real sample	Comments	Reference
PB modified nanoporous Au electrode	2.5	7.0	177 mA/M	–	good reproducibility; stable ≥60 days	Ahmadalinezhad et al.^63^
PB-Au nanostructures	8.8	5.8	9.9 mA/M	human serum	improved sensitivity and stability	Sakalauskiene et al.^64^
PB functionalized TiO_2_ Nanotube	3.2	6.0	248	human serum	–	Gao et al.^65^
PB entrapment in Nafion	1.0	7.4	1.59	–	decreased PB stability in pH 8.0	Haghighi et al.^66^
Macroporous polyaniline/PB	0.4	6.0	99.4	human serum	low detection limit of 0.4 μM	Chen et al.^67^
PB in ionic liquid-chitosan matrix	5.0	6.5	90.4	blood serum	wide linear-range (4.2 mM)	Zhang et al.^68^
PB in (PEDOT:PSS) conductive hydrogel	0.85	7.4	340.1	rabbit serum	good reproducibility	Xu et al.^69^
(PEDOT)/PB-CNT; post-deposition baking (100°C)	–	7.4	2.67	human serum	flexible screen-printed carbon electrode	Chiu et al.^70^
PB film tempered at 100°C	8.0	7.4	9.3 ± 0.1	brain extracellular fluid	10-μm carbon fiber electrodes; *in vivo* test	Salazar et al.^71^
PB/conductive ink	9.2	7.0	–	human sweat	flexible wearable biosensors	Blasques et al.^72^
PB/graphite strings	10	7.4	641.3	human serum	excellent reproducibility	Lee et al.^73^
N-doped graphene quantum dot modified PB analog	–	7.0	90.49 ± 1.08	human sweat	87% retention rate over long-term operation	Chiu et al.^74^
Reduced graphene oxide-PB film	8.0	7.4	27.78	cola and soda	wearable glucose sensors	Ma et al.^75^
PB nanoparticles synthesized on filter paper by evaporation	170	7.4	1.7 mA/M	blood samples	biosensors on screen-printed electrodes	Cinti et al.^76^
PB with stabilizing Ni hexacyanoferrate (layer-by-layer deposition)	1.0	6.0	230	undiluted sweat	non-invasive diabetes monitoring	Karpova et al.^77^

LOD, limit of detection; PEDOT, poly(3,4-ethylenedioxythiophene); PSS, polystyrene sulfonate.

### Metal ion-doping

Efforts have also been devoted to modifying the PB lattice by introducing metal ions (Ni, Co, or Mn) to form PB analogs, especially for battery electrode materials.^82^ Ni^2+^-doping has emerged as a popular candidate due to the distinct structural and thermodynamic merits. Structurally, its ionic radius closely aligns with that of high-spin Fe^3+^, permitting seamless isomorphous substitution or interstitial doping into the face-centered cubic lattice without introducing detrimental lattice distortion or phase segregation. Additionally, the integration of Ni^2+^ creates local isomorphic domains of nickel hexacyanoferrate, which possesses lower solubility and significantly higher chemical inertness toward hydroxyl ions than pure PB. Zhang et al. integrated Ni-Fe PB analog nanocubes with Au NPs for the continuous detection of H_2_O_2_ release from living cells in neutral solutions for more than 1 h, because Ni^2+^-doping and the gold interface enhanced the PB nanocube’s stability.^83^

While pure PB is most active for H_2_O_2_, mixed-metal hexacyanoferrates often exhibit superior structural integrity and operational stability in practical applications. For example, Zhang et al. found that the sensitivity of Mn-doping PB-based sensor decreased by 60% compared with the pristine PB at pH 3.0 but exhibited approximately the same level as PB at pH 7.4.^84^ Therefore, it is crucial to maintain the balance between total Fe ions and auxiliary metal ions for satisfying both catalytic-activity and stability.^77^ Incorporating approximately 30% of Ni^2+^ into the PB framework creates a more rigid lattice that is significantly more resistant to OH^−^ attack.^11^

Recently, molybdenum-doping PB has been frequently investigated in both H_2_O_2_ sensing and battery electrode materials.^85^^,^^86^ Wang et al. found the doping of molybdenum successfully tailored the size and morphology of PB-analog-based nanozymes, and its peroxidase-mimicking activity was enhanced by over 37-fold compared with that of pristine PB-analogs.^85^

### Other strategies

Additionally, some uncommon methods, such as pulse electrodeposition and post-deposition heating have been reported to improve PB stability to certain extents. For instance, 100 °C baking of the freshly prepared PB powder gives better stability in PBS (pH 7.4).^84^ Potential cycling in high-concentration KCl solutions has been reported to convert “insoluble” PB into a more stable “soluble” form by facilitating the intercalation of K^+^ ions that balance the lattice charge and prevent structural collapse^27^ (Figure 1).

## Oxidase-based biosensing

### Glucose biosensor

The evolution of PB in bioanalytical applications is inextricably linked to the development of glucose biosensors, which remain the most widely investigated electrochemical biosensors. The landmark demonstration of PB as an “artificial peroxidase” by Karyakin and colleagues in the 1990s revolutionized the landscape by enabling the highly sensitive detection of H_2_O_2_ at remarkably low overpotentials, typically near 0.0 V vs. Ag|AgCl. This was a monumental improvement over traditional noble-metal electrocatalysts like platinum and Au NPs, which required significantly higher potentials (e.g., > 0.5 V), leaving the biosensors vulnerable to interference from common electroactive species like ascorbic acid and uric acid.

By integrating GOx with PB-modified electrodes, researchers developed glucose biosensors that utilized the direct reduction of enzymatically produced H_2_O_2_ via the PB catalysis (Figure 5). This configuration endows glucose biosensors exceptional selectivity and sensitivity, lowering the detection limits into the micromolar range. Over the decades, many studies focused on engineering PB-based electrocatalysts for higher activity and durability in biosensing. We reviewed the most significant studies in the past two decades, and the studies that have tested real samples, such as serum, sweat, and soda drinks using their fabricated biosensors have been preferentially summarized in Table 1. Among them, the biosensor based on PB incorporated within the conductive hydrogel (PEDOT/PSS) obtained the highest sensitivity, 340.1 μA mM^−1^ cm^−2^ in PBS (pH 7.4), and the biosensors with good reproducibility have further been used to quantify glucose content in rabbit serum. PB and PB analogs have also been tested in the advancing wearable biosensors.^69^ For example, Chiu et al. incorporated N-doped graphene quantum dots into the protective layer containing PB analogs to develop wearable glucose biosensors, and the resulting biosensors’ sensitivity remained 88% of the newly fabricated one over long-term operation.^74^ This provided a promising solution for non-invasive glucose monitoring in human sweat and next-generation wearable biosensors for continuous diabetes management, although their electrode cost was remarkably higher than current test strips.

**Figure 5:**
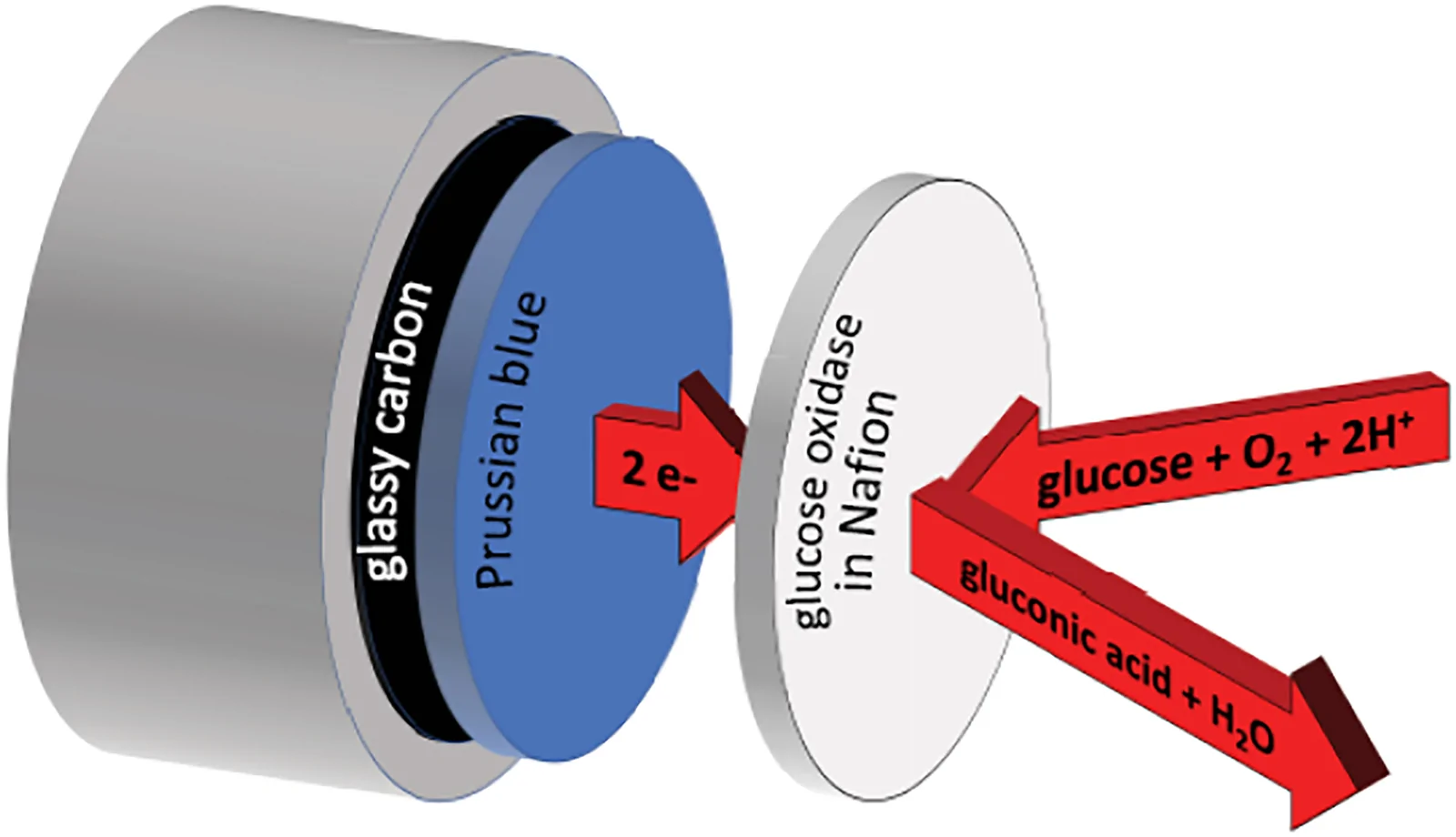
Schematic principle of a glucose biosensor based on PB-modified glassy carbon electrode with the Nafion coating Reproduced from Drew et al.,^24^*Journal of Chemical Education*, 2023.

### Other oxidase-based biosensors

The successful utilization of PB in glucose biosensors paved the way for its application across a broad spectrum of oxidase-based biosensors, establishing it as a universal transducer for any enzyme that consumes oxygen to generate H_2_O_2_ as a byproduct. For instance, lactate biosensors, essential for clinical diagnostics, sports physiology, and many other uses, were the most widely investigated biosensors beyond glucose biosensors. Smutok et al. employed PB-based lactate biosensors to determine the lactate content in wine (grape must) and other drinks.^87^

The recent focus has also transitioned to the integration of PB-oxidase platforms into wearable and point-of-care (POC) devices by utilizing the flexible, screen-printed electrodes, and microneedle arrays. For example, the development of skin-worn sweat sensors for the non-invasive monitoring of glucose and lactate has benefited immensely from the high catalytic efficiency of PB nanozymes, which allow for accurate measurements in the low-volume, fluctuating pH environment of human perspiration (Table 2).

**Table 2:** PB-based biosensors for other analytes

PB-based material (synthesis)	Analyte	Working pH	Sensitivity (mA/M cm^2^)	Real sample	Comments	Reference
PB on superhydrophobic carbon cloth	lactate	4.5, 5.5, 6.5, 7.5	22.28	undiluted sweat	wide linear range, up to 40 mM	Huang et al.^88^
PB deposited on carbon fibers	lactate	7.4	12	prefrontal cortex	anti-biofouling coating	Salazar et al.^89^
Pt nanoparticles modified graphite electrode	lactate	6.8	523	grape must and wine	good selectivity	Smutok et al.^87^
PB-(alkoxysilane -perfluorosulfonated ionomer) coating	lactate	7.0	200	–	catalytically synthesized PB	Vokhmyanina et al.^90^
PB within a nickel hexacyanoferrate shell	lactate	6.0	134 ± 12	–	steady-state currents decay ≥5 min	Pleshakov et al.^91^
A copolymer-graphene coating on PB	glutamate	7.0	3.96	neuronal cell	good stability and reproducibility	Rajarathinam et al.^92^
PB-polydopamine	glutamate	7.0	70.68	tomato juice	Au microelectrode	Lin et al.^93^
Graphene-PB nanocube/Au	glutamate	7.4	24	–	Au microelectrode	Chen et al.^58^
Graphene nanosheets-PB	sarcosine	8.0	9.04	human urine	–	Rajarathinam et al.^94^
PB-poly-(O-phenylenediamine)	galactose	7.0	127			Charmantray et al.^95^
PBA@Au edge-rich microcubes	sucrose	7.4	16.4 ± 0.4	alcohol fermentation	microchip biosensor with good reusability	Zhang et al.^96^
PB deposited on fluorine-doped tin oxide	urea	6.0	2.55 Ω cm^2^ mM^−1^	–	impedimetric biosensing	Valiūnienė et al.^97^
PB on sol-gel matrix	cholesterol	6.8	26.0	–	self-powered enzyme fuel cell	Sekretaryova et al.^98^
Graphene/PB composites	uric acid	6.0	5.14	–	one-pot synthesis	Dos Santos et al.^59^
CoFe-PBA	xanthine	6.0	105	urine, coffee, milk	–	Chu et al.^99^
PB/indium tin oxide nanoparticles	biogenic amines	7.4	1.84	spiked white cheese	screen-printed carbon electrodes	Kaçar et al.^100^

PBA, PB analogs.

Similarly, PB-based platforms have been adapted for the real-time monitoring of neurotransmitter fluctuations in brain tissues, such as glutamate and choline. Other notable applications include alcohol biosensors for controlling food quality and xanthine/hypoxanthine biosensors for assessing meat freshness (Table 2). In these systems, the optimization of the bio-interface between PB and enzyme layer for improved measurement accuracy has been one of the major technical challenges. For example, the use of chitosan-PB networks or PB-polypyrrole bilayers has successfully addressed the need for better stability while maintaining high ion permeability, ensuring that the biosensor remains responsive even when operating within complex biological matrices or viscous fluids.

## Emerging bioanalytical applications

### Intracellular H_2_O_2_ measurement

The development of PB-based electrochemical sensors into a specialized tool for biomedical research has been driven by the critical need for the precise, real-time, and *in situ* tracking of H_2_O_2_ dynamics in living cells. In biological systems, the elevated level of H_2_O_2_ is a hallmark of oxidative stress and a precursor to various pathologies, including neurodegeneration, inflammation, and cancer. The primary advantage of PB-based sensors in this context is their ability to operate at a cathodic potential near 0.0 V (vs. Ag|AgCl), which significantly minimizes the electrochemical oxidation of endogenous interference molecules within the cellular milieu. Unlike conventional optical assays, electrochemical detection can bypass the optical interference from endogenous cellular pigments and avoid complex cell treatment or labeling, allowing for the direct investigation of living cells in a physiological environment. However, their practical application is hindered by the complex extracellular environment, causing severe electrode surface fouling by proteins and cellular debris, leading to signal drift and reduced long-term stability. Overcoming these hurdles typically requires advanced surface modifications, such as the integration of permselective coatings or highly specific nanomaterial catalysts, to balance high biocompatibility with precise analytical performance.

For instance, recent studies have utilized PB-modified microelectrodes to monitor the H_2_O_2_ release from living cells such as macrophages and HeLa cells upon stimulation with phorbol myristate acetate (PMA) or cocoa polyphenols (Figure 6). Tumor cells often exhibit higher levels of intrinsic oxidative stress compared to healthy cells due to mitochondrial dysfunction and increased metabolic demands. PB-nanocomposites with improved activity and biocompatibility have been employed to detect H_2_O_2_ efflux in tumor cell lines (e.g., MCF-7 and HeLa) (Table 3). Electrochemical measurements of rapid release of H_2_O_2_ from living cells provide crucial data for understanding cellular responses to external stimuli, drug treatments, or pathological changes. However, it is still challenging to perform continuous measurement of cellular H_2_O_2_ level in the steady state due to the extremely low release rate in the extracellular. In the future, the utilization of electrochemical sensors as a reliable tool for specific studies in cell biology would be of great significance.

**Figure 6:**
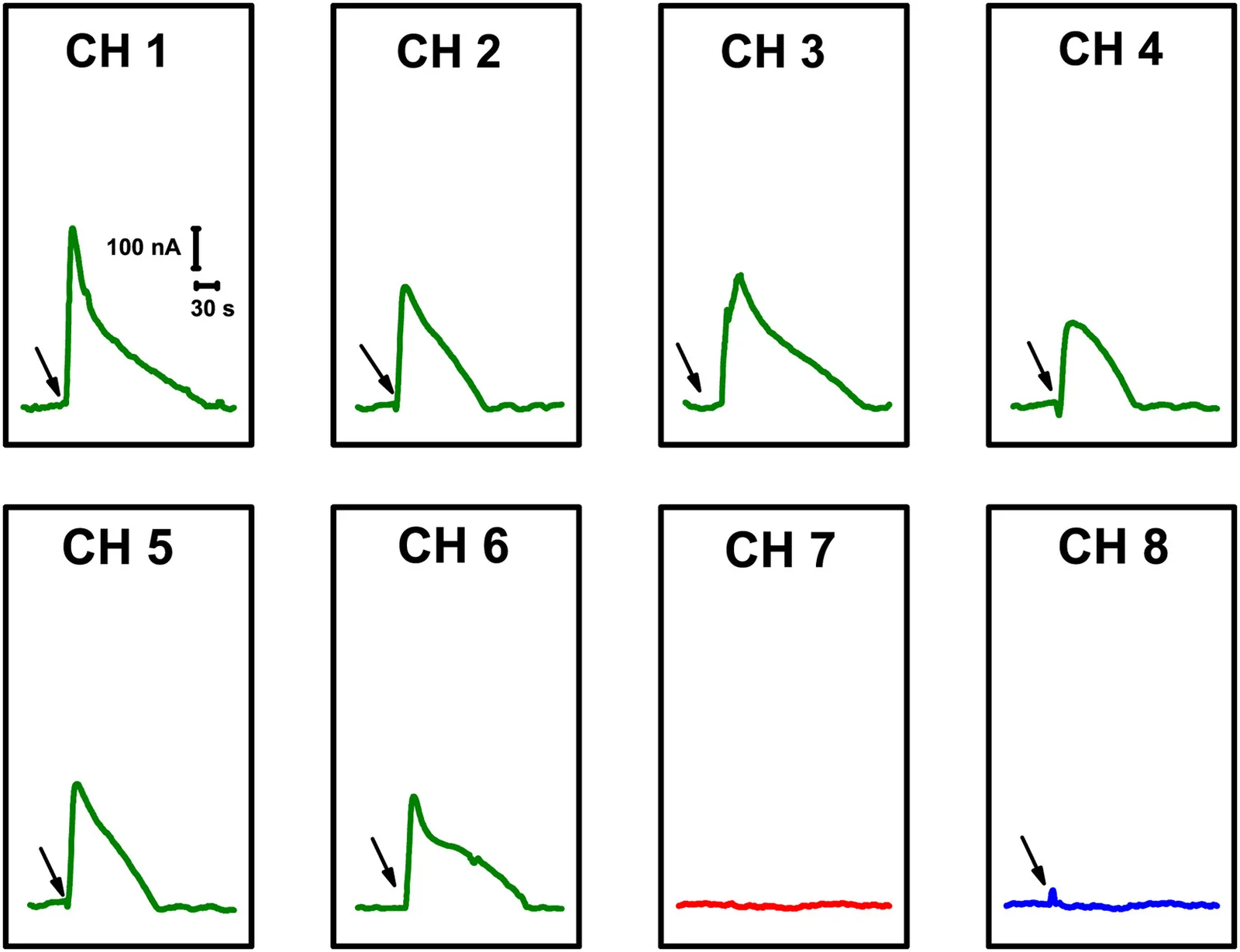
Amperometry simultaneous signals in the PB modified stencil-printed electrodes CH 1–6 shows typical responses for oxidative stress responses in HeLa cells after stimulation. CH 7 shows a negative control without adding stimulation agents. CH 8 shows the signal in the presence of 0.1 mg mL^−1^ catalase upon adding the stimulation agent. Arrows indicate the addition of 100 μM of cocoa polyphenols. X and Y scales are shown in CH 1 and are maintained in the rest of the channels. Reproduced from Rojas et al.,^101^*Biosensors and Bioelectronics*, 2020.

**Table 3:** PB-based electrochemical sensors applied for the determination of H_2_O_2_ released by cells

PB-based material (synthesis)	Working pH	LOD (μM)	Cell (tissue)	Stimulation agent	Comments	Reference
PB modified electrode array	7.4	1.9	HeLa cells	cocoa polyphenols	printed electrode on PET	Rojas et al.^101^
PB on electrochemically reduced graphene oxide	7.4	0.8	macrophage cells	lipopolysaccharide	–	Qiu et al.^102^
PB/graphenated carbon nanotubes	6.8	0.013	RAW 264.7 cells	lipopolysaccharide	–	Balamurugan et al.^15^
PB modified carbon black with Nafion coating	7.4	0.01	neuroblastoma cell	6-hydroxydopamine, a neurotoxin	Parkinson’s disease model	Rojas et al.^103^
PB-carbon nanotube composite	7.4	1.0	cancer cells	ascorbic acid	microneedle electrode	Zhu et al.^104^
Polydopamine-PB coated carbon fiber	*in vivo*	0.4	cortex of rats	mercaptosuccinate; electrical stimulation	good selectivity	Li et al.^105^

### Electrochemical immunosensing

Beyond sensing H_2_O_2_, PB also serves as a probe for label-free electrochemical immunosensing in two distinct modes.^106^ Firstly, PB is typically electrodeposited onto the electrode surface with immobilized antibody like enzyme layer to provide a baseline redox current. When the target antigen binds to the antibody on electrode, it forms a bulky, non-conductive immunocomplex. This biomolecular complex acts as a physical barrier (steric hindrance) that inhibits the diffusion of counter-ions (K^+^) or electrons between the PB lattice and the electrolyte. Therefore, the peak current of the PB redox couple decreases proportionally to the antigen concentrations. This approach is widely used for its simplicity and label-free merits. For example, an Au NPs/PB-poly(3,4-ethylenedioxythiophene) nanocomposite was employed to detect carcinoembryonic antigen at concentration as low as 0.01 ng mL^−1^ in PBS (pH 6.0).^79^ Recently, Kongkaew et al. modified a multi-electrode array with PB and specific antibodies to detect four breast cancer tumor markers simultaneously.^107^

Secondly, PB is used as a labeling agent, often in the form of dispersed nanoparticles (or nanozymes) conjugated to a detection antibody within a sandwich-type assay. When in the presence of target antigens, the sandwich complex is formed, leading to the increase in the redox current of PB. Since the captured PB-labeled antibody is proportional to the antigen concentration, the current signal increases accordingly. This design significantly improves immunosensing sensitivity and lowers the limit of detection (LOD) compared to label-free methods. For example, PB-based sandwich immunosensing assay has been employed to detect glycated albumin,^108^ alpha-fetoprotein,^109^ and avian influenza virus.^110^ The material stability of PB is especially crucial to the accurate and reliable immunosensing because it largely determines the constant baseline signal during the antigen-antibody binding process. Additionally, the biofouling and non-specific protein adsorption should be eliminated when testing practical samples.

### Peroxidase kinetics assays

Lignocellulose-degrading enzymes are essential to fungal physiology and are increasingly valued in industrial applications such as biofuel production, environmental bioremediation, and pulp processing. However, acquiring their accurate kinetic data on insoluble lignocellulose substrates remains challenging using traditional spectrophotometric, fluorometric, or calorimetric assays, therefore hindering enzyme discovery and engineering efforts.^111^ To address this challenge, PB-based H_2_O_2_ sensor has emerged as a powerful tool for investigating lignin-degrading peroxidases and lytic polysaccharide monooxygenases (LPMOs, also functioning as peroxygenases).^17^^,^^49^ The main advantage of the electrochemical assay lies in its inherent immunity to the turbidity and coloration inherent in complex and heterogeneous substrate suspensions, enabling direct and precise monitoring of H_2_O_2_ dynamics during enzymatic degradation of insoluble substrates.

For instance, PB-modified electrodes were successfully employed to monitor the *in situ* activity of lignin peroxidase from *Amycolatopsis* while degrading insoluble kraft lignin (Figure 7). Similarly, this approach was adapted to investigate LPMOs, which utilize H_2_O_2_ as a co-substrate to break crystalline cellulose particles and cellulose embedded in wood-cell-walls.^112^ The use of PB transducers allowed researchers to directly measure the consumption rates on solid cellulose surfaces, resolving ambiguities regarding their oxygen-versus-peroxide dependency during heterogeneous catalysis. Considering the ubiquity of peroxidases across biological systems and their critical roles in diverse industries, PB-based H_2_O_2_ sensors are expected to transition from fundamental kinetics studies to high-throughput screening of enzyme libraries engineered for harsh industrial conditions. For examples, the antioxidative systems in plants, which mainly include catalase, ascorbate peroxidase, and glutathione peroxidases, tightly control the cellular H_2_O_2_ levels in overcoming various environmental stress.^113^^,^^114^ This PB-based H_2_O_2_ sensor has shown great potential in quantifying the kinetics of these crude H_2_O_2_-scavenging enzymes.

**Figure 7:**
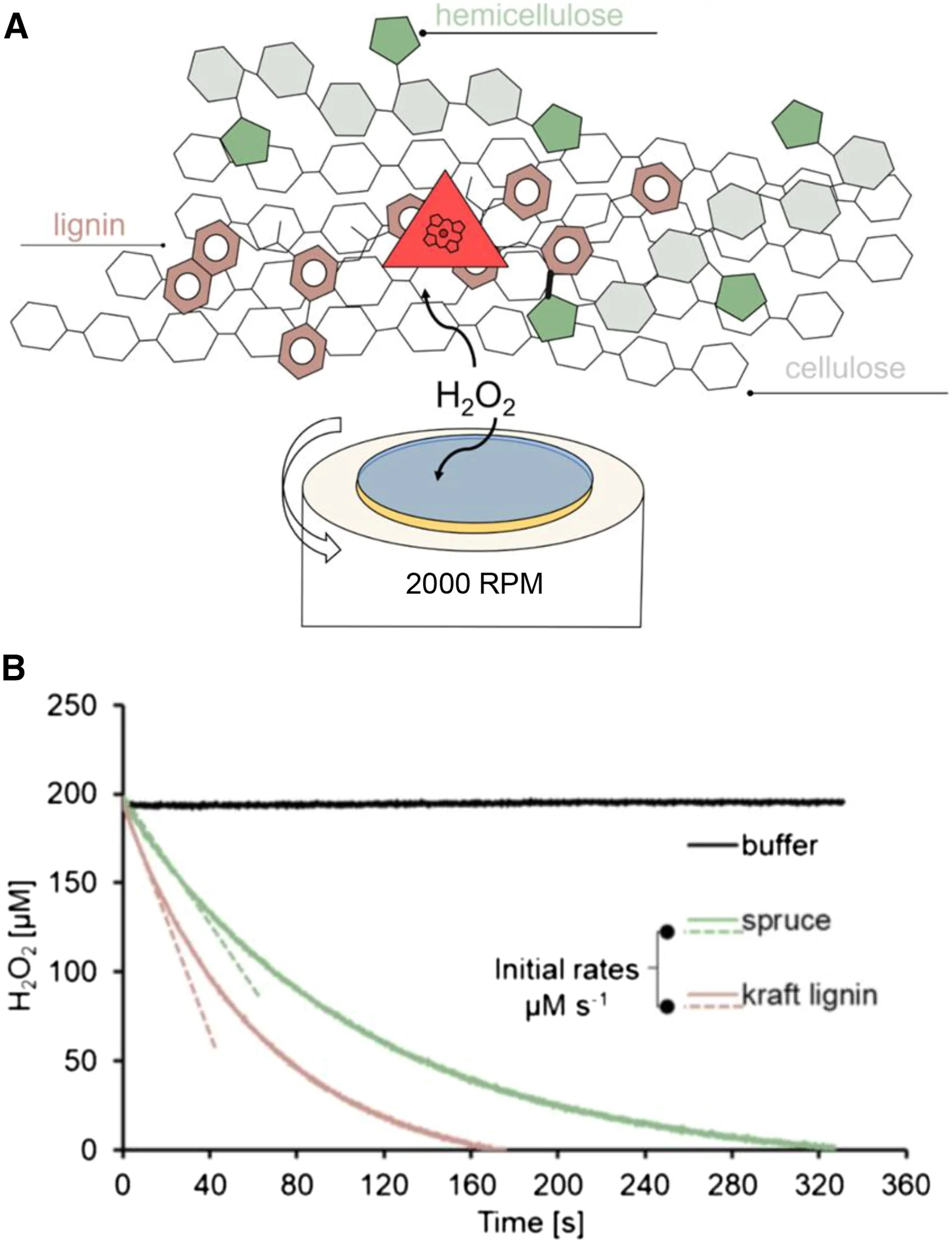
PB-based H_2_O_2_ sensor for assaying peroxidase kinetics (A) Schematic principle of the electrochemical assay for measuring kinetics of lignin peroxidase. The H_2_O_2_ sensor was fabricated by modifying a gold rotating disc electrode with PB films. (B) H_2_O_2_ consumption curves of bacterial dye-decolorizing peroxidase on kraft lignin or spruce as substrate. The initial H_2_O_2_ consumption rates are determined using linear regression from 0 to 40 s from the starting point. Reproduced with permission from Välimets et al.,^49^*ACS Omega*, 2024.

## Conclusion and outlook

This review has outlined the chemical and structural fundamentals of PB, detailing synthesis strategies and the pH-sensitive instability of PB electrocatalysts across bioanalytical platforms ranging from oxidase biosensors to universal peroxidase kinetic assays.

Moving forward, the true trajectory of PB-based bioanalysis lies in transitioning from idealized laboratory setups to the diversified settings for practical deployment. Future application perspectives could prioritize the integration of highly stabilized PB frameworks into flexible, wearable POC devices for continuous, non-invasive monitoring of sweat or interstitial fluids. Furthermore, exploiting PB’s exceptional low-potential electroanalysis will open new avenues for tracking extracellular signaling and transient oxidative stress dynamics in living cells. Ultimately, overcoming stability limitations through rational hybrid nanocomposite engineering and protective encapsulation will bridge the gap between proof-of-concept research and translation technology of clinical diagnostics.

## Acknowledgments

This work was funded by the 10.13039/501100001809National Natural Science Foundation of China (32301048) and the 10.13039/501100007129Shandong Provincial Natural Science Foundation (2024HWYQ-012). This work was also funded by the Startup Funding for Young Scholars at Changzhi University.

## Author contributions

Conceptualization, H.C. and S.M.; writing – original draft, H.C. and S.M.; writing – making tables, H.C., J.H., Yuhang Zhang, Y.L., Yan Zhang, and T.W.; writing – review and editing, H.C., Q.T., J.H., Yuhang Zhang, Y.L., Yan Zhang, T.W. and J.-J.D.; funding acquisition, H.C. and S.M.; supervision, H.C.

## Declaration of interests

The authors declare no competing interests.

## References

[1] Samain L., Grandjean F., Long G.J., Martinetto P., Bordet P., Strivay D. (2013). Relationship between the synthesis of prussian blue pigments, their color, physical properties, and their behavior in paint layers. J. Phys. Chem. C.

[2] Neff V.D. (1978). Electrochemical Oxidation and Reduction of Thin Films of Prussian Blue. J. Electrochem. Soc..

[3] Komkova M.A., Karyakina E.E., Karyakin A.A. (2018). Catalytically Synthesized Prussian Blue Nanoparticles Defeating Natural Enzyme Peroxidase. J. Am. Chem. Soc..

[4] Komkova M.A., Karyakin A.A. (2022). Prussian blue: from advanced electrocatalyst to nanozymes defeating natural enzyme. Mikrochim. Acta.

[5] Xu M., Yu Z., Wu D., Lao S., Sheng E., Tang D. (2025). Smartphone-Integrated Device Based on d-Band Engineered PtNiFe Nanozymes for on-Site Testing of Perfluorinated Substances. Anal. Chem..

[6] Xu M., Yu Z., Lao S., Sheng E., Tang D. (2025). Bifunctional Block Polymer-Engineered Platinum Nanozymes for Ultrasensitive Immunoassay. Anal. Chem..

[7] Lundgren C.A., Murray R.W. (1988). Observations on the composition of Prussian blue films and their electrochemistry. Inorg. Chem..

[8] Malinauskas A., Araminait≐ R., Mickevičiūt≐ G., Garjonyt≐ R. (2004). Evaluation of operational stability of Prussian blue- and cobalt hexacyanoferrate-based amperometric hydrogen peroxide sensors for biosensing application. Mater. Sci. Eng. C.

[9] Karyakin A.A., Karyakina E.E., Gorton L. (1999). On the mechanism of H_2_O_2_ reduction at Prussian Blue modified electrodes. Electrochem. Commun..

[10] Ming H., Zhang Y., Wang H., Wang S., Gong H., Pan J., Zhang H., Zhang B., Xia J., Sun J. (2025). In Situ Electrochemical Incorporation of La^3+^ into Prussian Blue Frameworks: A Facile Strategy for Enhanced Structural Integrity and Crystalline Water Management. Small.

[11] Lin D., Hua R., Yang H., Xu C., Yang S., Chen J., Qu D., Li X., Liu D. (2025). Improving the electrochemical performance of Prussian-Blue-analogue cathode by the co-doping of cobalt and nickel and its application in aqueous hybrid ions battery. Electrochim. Acta.

[12] Lee S.Y., Park J.-Y., Kim H.-J., Lee Y.-S., Park Y.I. (2022). Prussian blue-graphene oxide composite cathode for a sodium-ion capacitor with improved cyclic stability and energy density. J. Alloys Compd..

[13] Talagaeva N.V., Kleinikova S.A., Gor’kov K.V., Zolotukhina E.V. (2020). Electrochemical and electrocatalytic stability of Prussian blue/Berlin green redox transformation in Prussian blue-polypyrrole composite films. J. Solid State Electrochem..

[14] Karpova E.V., Shcherbacheva E.V., Komkova M.A., Eliseev A.A., Karyakin A.A. (2021). Core–Shell Nanozymes “Artificial Peroxidase”: Stability with Superior Catalytic Properties. J. Phys. Chem. Lett..

[15] Balamurugan T.S.T., Mani V., Hsieh C.-C., Huang S.-T., Peng T.-K., Lin H.-Y. (2018). Real-time tracking and quantification of endogenous hydrogen peroxide production in living cells using graphenated carbon nanotubes supported Prussian blue cubes. Sensor. Actuator. B Chem..

[16] Babu S., Lee K., Yang H. (2024). Enzymatic precipitation of highly electroactive and ion-transporting prussian blue for a sensitive electrochemical immunosensor. ACS Sens..

[17] Schwaiger L., Csarman F., Chang H., Golten O., Eijsink V.G.H., Ludwig R. (2024). Electrochemical monitoring of heterogeneous peroxygenase reactions unravels LPMO kinetics. ACS Catal..

[18] Keggin J.F., Miles F.D. (1936). Structures and Formulæ of the Prussian Blues and Related Compounds. Nature.

[19] Kraft A. (2021). Some considerations on the structure, composition, and properties of Prussian blue: a contribution to the current discussion. Ionics.

[20] Robin M.B. (1962). The Color and Electronic Configurations of Prussian Blue. Inorg. Chem..

[21] Piernas Muñoz M.J., Piernas Muñoz M.J., Castillo Martínez E. (2018). Prussian Blue Based Batteries.

[22] Kulesza P.J., Doblhofer K. (1989). The membrane properties of Prussian Blue films on electrodes. J. Electroanal. Chem. Interfacial Electrochem..

[23] Karyakin A.A., Karyakina E.E. (1999). Prussian Blue-based 'artificial peroxidase' as a transducer for hydrogen peroxide detection. Application to biosensors. Sensor. Actuator. B Chem..

[24] Drew S.M., Belzer T. (2023). An amperometric glucose biosensor composed of prussian blue, Nafion, and glucose oxidase studied by flow injection analysis. J. Chem. Educ..

[25] Zeng R., Huang Z., Wang Y., Tang D. (2020). Enzyme-encapsulated DNA hydrogel for highly efficient electrochemical sensing glucose. Chemelectrochem.

[26] Dong H., Wang G., Feng K., Wu X., Fan Y., Zhang W., Ma M., Gu N., Zhang Y. (2022). Reference material of Prussian blue nanozymes for their peroxidase-like activity. Analyst.

[27] Avila L.B., Müller C.K., Hildebrand D., Faita F.L., Baggio B.F., Cid C.C.P., Pasa A.A. (2020). Resistive Switching in Electrodeposited Prussian Blue Layers. Materials.

[28] Uemura T., Kitagawa S. (2003). Prussian Blue Nanoparticles Protected by Poly(vinylpyrrolidone). J. Am. Chem. Soc..

[29] Peng L., Gineste S., Coudret C., Ciuculescu-Pradines D., Benoît-Marquié F., Mingotaud C., Marty J.-D. (2023). Iron-based hybrid polyionic complexes as chemical reservoirs for the pH-triggered synthesis of Prussian blue nanoparticles. J. Colloid Interface Sci..

[30] Zakaria M.B., Chikyow T. (2017). Recent advances in Prussian blue and Prussian blue analogues: synthesis and thermal treatments. Coord. Chem. Rev..

[31] Nai J., Lou X.W.D. (2019). Hollow Structures Based on Prussian Blue and Its Analogs for Electrochemical Energy Storage and Conversion. Adv. Mater..

[32] Yu Z., Xu M., Xu M., Gu J., Xue H., Tang D. (2026). Fe–N_3_S Single-Atom Nanozyme with Asymmetric Coordination for Ultra-Low-Background Colorimetric Immunoassays. Anal. Chem..

[33] Lao S., Gao F., Yu Z., Xu M., Sheng E., Tie D., He Y., Tang D. (2026). Partially Phosphidated Prussian Blue Analogues Derived Heterostructure Nanozyme for Colorimetric-Photothermal Dual-Mode Immunoassay. Anal. Chem..

[34] Ming H., Torad N.L.K., Chiang Y.-D., Wu K.C.W., Yamauchi Y. (2012). Size- and shape-controlled synthesis of Prussian Blue nanoparticles by a polyvinylpyrrolidone-assisted crystallization process. CrystEngComm.

[35] Hornok V., Dékány I. (2007). Synthesis and stabilization of Prussian blue nanoparticles and application for sensors. J. Colloid Interface Sci..

[36] Shiba F., Mameuda U., Tatejima S., Okawa Y. (2019). Synthesis of uniform Prussian blue nanoparticles by a polyol process using a polyethylene glycol aqueous solution. RSC Adv..

[37] Shiba F. (2010). Preparation of monodisperse Prussian blue nanoparticles via reduction process with citric acid. Colloids Surf. A Physicochem. Eng. Asp..

[38] Wu J., Yin F. (2013). Sensitive enzymatic glucose biosensor fabricated by electrospinning composite nanofibers and electrodepositing Prussian blue film. J. Electroanal. Chem..

[39] Haghighi B., Varma S., Alizadeh Sh F.M., Yigzaw Y., Gorton L. (2004). Prussian blue modified glassy carbon electrodes—study on operational stability and its application as a sucrose biosensor. Talanta.

[40] De Mattos I.L., Gorton L., Ruzgas T. (2003). Sensor and biosensor based on Prussian Blue modified gold and platinum screen printed electrodes. Biosens. Bioelectron..

[41] Yu Z., Xu Z., Zeng R., Xu M., Zou M., Huang D., Weng Z., Tang D. (2025). Tailored Metal–Organic Framework-Based Nanozymes for Enhanced Enzyme-Like Catalysis. Angew. Chem. Int. Ed. Engl..

[42] Karpova E.V., Karyakina E.E., Karyakin A.A. (2017). Communication—Accessing Stability of Oxidase-Based Biosensors via Stabilizing the Advanced H_2_O_2_ Transducer. J. Electrochem. Soc..

[43] Sterzinger J., Streng R., Chen S., Götz R., Hu W., Li J., Bandarenka A.S. (2025). Degradation Mechanisms of Prussian Blue Analogues and State-of-the-Art Approaches for Stability Optimization: A Review. J. Phys. Chem. C.

[44] Péter T., Takács D., Viczián D., Katana B., Alsharif N.B., Szilagyi I. (2025). Correlations Between Colloidal Stability and Peroxidase Activity of Prussian Blue Nanozymes in Salt Solutions. J. Phys. Chem. B.

[45] Noël J.-M., Médard J., Combellas C., Kanoufi F. (2016). Prussian Blue Degradation during Hydrogen Peroxide Reduction: A Scanning Electrochemical Microscopy Study on the Role of the Hydroxide Ion and Hydroxyl Radical. Chemelectrochem.

[46] Gervais C., Languille M.-A., Réguer S., Gillet M., Pelletier S., Garnier C., Vicenzi E.P., Bertrand L. (2013). Why does Prussian blue fade? Understanding the role(s) of the substrate. J. Anal. At. Spectrom..

[47] Gu H., Lai Z., Wang X., Sun W., Rong Y., Guo F. (2025). Exploring the stabilization effect and mechanism of Nafion coating on Prussian blue analogue electrodes. J. Alloys Compd..

[48] Ahn J., Kim S., Jeon S.-I., Lee C., Lee J., Yoon J. (2021). Nafion-coated Prussian blue electrodes to enhance the stability and efficiency of battery desalination system. Desalination.

[49] Välimets S., Schwaiger L., Bennett A., Maresch D., Ludwig R., Hann S., Linde D., Ruiz-Dueñas F.J., Peterbauer C. (2024). Dye-Decolorizing Peroxidases Maintain High Stability and Turnover on Kraft Lignin and Lignocellulose Substrates. ACS Omega.

[50] Berkheimer Z.A., Tahir A., Nordin G.P., Paixão T.R.L.C., Woolley A.T., do Nascimento G.H.M., de Araujo W.R., Pradela-Filho L.A. (2025). Extruded filament electrodes for lactate biosensing in continuous-injection paper-based microfluidic devices. Biosens. Bioelectron..

[51] Garjonyte R., Yigzaw Y., Meskys R., Malinauskas A., Gorton L. (2001). Prussian Blue- and lactate oxidase-based amperometric biosensor for lactic acid. Sensor. Actuator. B Chem..

[52] Jirakunakorn R., Khumngern S., Choosang J., Thavarungkul P., Kanatharana P., Numnuam A. (2020). Uric acid enzyme biosensor based on a screen-printed electrode coated with Prussian blue and modified with chitosan-graphene composite cryogel. Microchem. J..

[53] Kim D.H., Lee H.B., Ha C.H., Jeon S.M., Seong G.H. (2025). Chitosan-stabilized Prussian blue nanoparticle-based multi-mode readout colorimetric, fluorometric, and photothermal biosensor for sensitive uric acid detection. Microchem. J..

[54] Wang X., Gu H., Yin F., Tu Y. (2009). A glucose biosensor based on Prussian blue/chitosan hybrid film. Biosens. Bioelectron..

[55] Barber R., Davis J., Papakonstantinou P. (2023). Stable Chitosan and Prussian Blue-Coated Laser-Induced Graphene Skin Sensor for the Electrochemical Detection of Hydrogen Peroxide in Sweat. ACS Appl. Nano Mater..

[56] Baretta R., Raucci A., Cinti S., Frasconi M. (2023). Porous hydrogel scaffolds integrating Prussian Blue nanoparticles: A versatile strategy for electrochemical (bio)sensing. Sensor. Actuator. B Chem..

[57] Lee G.-G., Hong H.-G. (2023). Catalytically Synthesized Prussian Blue by a One-Step Copolymerization of Polydopamine-Polypyrrole for Electrochemical Sensing of Hydrogen Peroxide. Electrochim. Acta.

[58] Chen J., Yu Q., Fu W., Chen X., Zhang Q., Dong S., Chen H., Zhang S. (2020). A Highly Sensitive Amperometric Glutamate Oxidase Microbiosensor Based on a Reduced Graphene Oxide/Prussian Blue Nanocube/Gold Nanoparticle Composite Film-Modified Pt Electrode. Sensors (Basel).

[59] Dos Santos P.L., Katic V., Toledo K.C.F., Bonacin J.A. (2018). Photochemical one-pot synthesis of reduced graphene oxide/Prussian blue nanocomposite for simultaneous electrochemical detection of ascorbic acid, dopamine, and uric acid. Sensor. Actuator. B Chem..

[60] Du D., Wang M., Qin Y., Lin Y. (2010). One-step electrochemical deposition of Prussian Blue–multiwalled carbon nanotube nanocomposite thin-film: preparation, characterization and evaluation for H2O2 sensing. J. Mater. Chem..

[61] Xu M., Yu Z., Wei Q., Tang D. (2025). Inflammatory microenvironment-responsive electrochemical biosensing for cancer cell discrimination using PtZnCd-anchored multi-walled carbon nanotubes. Talanta.

[62] Zou Y., Sun L.-X., Xu F. (2007). Biosensor based on polyaniline–Prussian Blue/multi-walled carbon nanotubes hybrid composites. Biosens. Bioelectron..

[63] Ahmadalinezhad A., Kafi A.K.M., Chen A. (2009). Glucose biosensing based on the highly efficient immobilization of glucose oxidase on a Prussian blue modified nanostructured Au surface. Electrochem. Commun..

[64] Sakalauskiene L., Brasiunas B., Popov A., Kausaite-Minkstimiene A., Ramanaviciene A. (2023). The Development of Reagentless Amperometric Glucose Biosensor Based on Gold Nanostructures, Prussian Blue and Glucose Oxidase. Biosensors.

[65] Gao Z.-D., Qu Y., Li T., Shrestha N.K., Song Y.-Y. (2014). Development of Amperometric Glucose Biosensor Based on Prussian Blue Functionlized TiO_2_ Nanotube Arrays. Sci. Rep..

[66] Haghighi B., Hamidi H., Gorton L. (2010). Electrochemical behavior and application of Prussian blue nanoparticle modified graphite electrode. Sensor. Actuator. B Chem..

[67] Chen X., Chen Z., Tian R., Yan W., Yao C. (2012). Glucose biosensor based on three dimensional ordered macroporous self-doped polyaniline/Prussian blue bicomponent film. Anal. Chim. Acta.

[68] Zhang Y., Liu Y., Chu Z., Shi L., Jin W. (2013). Amperometric glucose biosensor based on direct assembly of Prussian blue film with ionic liquid-chitosan matrix assisted enzyme immobilization. Sensor. Actuator. B Chem..

[69] Xu C., Jiang D., Ge Y., Huang L., Xiao Y., Ren X., Liu X., Zhang Q., Wang Y. (2022). A PEDOT:PSS conductive hydrogel incorporated with Prussian blue nanoparticles for wearable and noninvasive monitoring of glucose. Chem. Eng. J..

[70] Chiu J.Y., Yu C.M., Yen M.J., Chen L.C. (2009). Glucose sensing electrodes based on a poly(3,4-ethylenedioxythiophene)/Prussian blue bilayer and multi-walled carbon nanotubes. Biosens. Bioelectron..

[71] Salazar P., Martín M., Roche R., González-Mora J.L., O'Neill R.D. (2010). Microbiosensors for glucose based on Prussian Blue modified carbon fiber electrodes for in vivo monitoring in the central nervous system. Biosens. Bioelectron..

[72] Blasques R.V., Stefano J.S., Camargo J.R., Guterres e Silva L.R., Brazaca L.C., Janegitz B.C. (2022). Disposable Prussian blue-anchored electrochemical sensor for enzymatic and non-enzymatic multi-analyte detection. Sens. Actuators Rep..

[73] Lee S.H., Chung J.-H., Park H.-K., Lee G.-J. (2016). A Simple and Facile Glucose Biosensor Based on Prussian Blue Modified Graphite String. J. Sens..

[74] Chiu Y.-H., Rinawati M., Chang L.-Y., Aulia S., Li C., Shi P.-C., Chen K.-J., Huang W.-H., Mizuguchi H., Yeh M.-H. (2025). Promoting the signal reliability of non-invasive biosensors via a N-doped graphene quantum dot modified Prussian blue analogue protective layer for glucose monitoring. J. Mater. Chem. B.

[75] Ma J., Du Y., Jiang Y., Shen L., Ma H., Lv F., Cui Z., Pan Y., Shi L., Zhu N. (2022). Wearable healthcare smart electrochemical biosensors based on co-assembled prussian blue—graphene film for glucose sensing. Mikrochim. Acta.

[76] Cinti S., Cusenza R., Moscone D., Arduini F. (2018). Paper-based synthesis of Prussian Blue Nanoparticles for the development of whole blood glucose electrochemical biosensor. Talanta.

[77] Karpova E.V., Shcherbacheva E.V., Galushin A.A., Vokhmyanina D.V., Karyakina E.E., Karyakin A.A. (2019). Noninvasive Diabetes Monitoring through Continuous Analysis of Sweat Using Flow-Through Glucose Biosensor. Anal. Chem..

[78] Cai G., Yu Z., Ren R., Tang D. (2018). Exciton–Plasmon Interaction between AuNPs/Graphene Nanohybrids and CdS Quantum Dots/TiO_2_ for Photoelectrochemical Aptasensing of Prostate-Specific Antigen. ACS Sens..

[79] Yang T., Gao Y., Liu Z., Xu J., Lu L., Yu Y. (2017). Three-dimensional gold nanoparticles/prussian blue-poly(3,4-ethylenedioxythiophene) nanocomposite as novel redox matrix for label-free electrochemical immunoassay of carcinoembryonic antigen. Sensor. Actuator. B Chem..

[80] Ghaderi S., Mehrgardi M.A. (2014). Prussian blue-modified nanoporous gold film electrode for amperometric determination of hydrogen peroxide. Bioelectrochemistry.

[81] Jiang X., Pan C., Wang Q., Han X., Tang D. (2023). Photoelectrochemical immunoassay for thyroglobulin on nanogold-functionalized BiVO_4_ photoanode coupling with enzymatic biocatalytic precipitation. Anal. Chim. Acta.

[82] Xu Z., Sun Y., Xie J., Nie Y., Xu X., Tu J., Zhang J., Qiu L., Zhu T., Zhao X. (2022). Scalable Preparation of Mn/Ni Binary Prussian Blue as Sustainable Cathode for Harsh-Condition-Tolerant Sodium-Ion Batteries. ACS Sustain. Chem. Eng..

[83] Zhang W., Wang C., Guan L., Peng M., Li K., Lin Y. (2019). A non-enzymatic electrochemical biosensor based on Au@PBA(Ni–Fe):MoS_2_ nanocubes for stable and sensitive detection of hydrogen peroxide released from living cells. J. Mater. Chem. B.

[84] Zhang Y., Kudriashov D., Pershina L., Offenhäusser A., Mourzina Y. (2022). Intrinsic Multienzyme-like Activities of the Nanoparticles of Mn and Fe Cyano-Bridged Assemblies. Nanomaterials.

[85] Wang C., Ren G., Yuan B., Zhang W., Lu M., Liu J., Li K., Lin Y. (2020). Enhancing Enzyme-like Activities of Prussian Blue Analog Nanocages by Molybdenum Doping: Toward Cytoprotecting and Online Optical Hydrogen Sulfide Monitoring. Anal. Chem..

[86] Wang S., Lei Y., Wang G., Zhao L., Shen X., Li S., Du S., Yang C., Qiu J. (2025). High Performance Capacitive Deionization Cathode of Nickel Hexacyanoferrate Doped with Trace Molybdenum: Breaking the Capacity-Stability Trade-Off. Angew. Chem. Int. Ed. Engl..

[87] Smutok O., Kavetskyy T., Prokopiv T., Serkiz R., Šauša O., Novák I., Švajdlenková H., Maťko I., Gonchar M., Katz E. (2022). Biosensor Based on Peroxidase-Mimetic Nanozyme and Lactate Oxidase for Accurate L-Lactate Analysis in Beverages. Biosensors.

[88] Huang L., Zeng M., Li Y., Liu Z., Zhang J., Feng X. (2025). Solid/Liquid/Gas Three-Phase Interface Enzymatic Reaction-Based Lactate Biosensor with Simultaneously High Sensitivity and Wide Linear Range. ACS Sens..

[89] Salazar P., Martin M., O’Neill R.D., Roche R., Gonzalez-Mora J.L. (2012). Biosensors Based On Prussian Blue Modified Carbon Fibers Electrodes for Monitoring Lactate in The Extracellular Space of Brain Tissue. Int. J. Electrochem. Sci..

[90] Vokhmyanina D.V., Andreeva K.D., Komkova M.A., Karyakina E.E., Karyakin A.A. (2020). ‘Artificial peroxidase’ nanozyme - enzyme based lactate biosensor. Talanta.

[91] Pleshakov V., Daboss E., Karyakin A. (2023). Novel Electrochemical Lactate Biosensors Based on Prussian Blue Nanoparticles. Eng. Proc..

[92] Rajarathinam T., Thirumalai D., Jayaraman S., Yang S., Ishigami A., Yoon J.-H., Paik H.-J., Lee J., Chang S.-C. (2024). Glutamate oxidase sheets-Prussian blue grafted amperometric biosensor for the real time monitoring of glutamate release from primary cortical neurons. Int. J. Biol. Macromol..

[93] Lin Y., Ma Y., Ye J. (2024). A modified Prussian blue biosensor with improved stability based on the use of self-assembled monolayers and polydopamine for quantitative L-glutamate detection. Mikrochim. Acta.

[94] Rajarathinam T., Kwon M., Thirumalai D., Kim S., Lee S., Yoon J.-H., Paik H.-J., Kim S., Lee J., Ha H.K., Chang S.C. (2021). Polymer-dispersed reduced graphene oxide nanosheets and Prussian blue modified biosensor for amperometric detection of sarcosine. Anal. Chim. Acta.

[95] Charmantray F., Touisni N., Hecquet L., Noguer T., Mousty C. (2015). Galactose Oxidase/Prussian Blue Based Biosensors. Electroanalysis.

[96] Zhang K., Wang M., Liu T., Chu Z., Jin W. (2022). Scalable Printing of Prussian Blue Analogue@Au Edge-Rich Microcubes as Flexible Biosensing Microchips Performing Ultrasensitive Sucrose Fermentation Monitoring. ACS Appl. Mater. Interfaces.

[97] Valiūnienė A., Kavaliauskaitė G., Virbickas P., Ramanavičius A. (2021). Prussian blue based impedimetric urea biosensor. J. Electroanal. Chem..

[98] Sekretaryova A.N., Beni V., Eriksson M., Karyakin A.A., Turner A.P.F., Vagin M.Y. (2014). Cholesterol Self-Powered Biosensor. Anal. Chem..

[99] Chu M., Wang Y., Xin J., Zhang L., Liu Y., Yang G., Ma H., Wang Y., Pang H., Wang X. (2023). In situ growth of CoFe-prussian blue analog nanospheres on ferrocene-functionalized ultrathin layered Ti_3_C_2_T_x_ MXene frameworks for efficient detection of xanthine. Chem. Eng. J..

[100] Kaçar C., Erden P.E., Dalkiran B., İnal E.K., Kiliç E. (2020). Amperometric biogenic amine biosensors based on Prussian blue, indium tin oxide nanoparticles and diamine oxidase– or monoamine oxidase–modified electrodes. Anal. Bioanal. Chem..

[101] Rojas D., Hernández-Rodríguez J.F., Della Pelle F., Del Carlo M., Compagnone D., Escarpa A. (2020). Oxidative stress on-chip: Prussian blue-based electrode array for in situ detection of H_2_O_2_ from cell populations. Biosens. Bioelectron..

[102] Qiu W., Zhu Q., Gao F., Gao F., Huang J., Pan Y., Wang Q. (2017). Graphene oxide directed in-situ synthesis of Prussian blue for non-enzymatic sensing of hydrogen peroxide released from macrophages. Mater. Sci. Eng. C Mater. Biol. Appl..

[103] Rojas D., Della Pelle F., Del Carlo M., d’Angelo M., Dominguez-Benot R., Cimini A., Escarpa A., Compagnone D. (2018). Electrodeposited Prussian Blue on carbon black modified disposable electrodes for direct enzyme-free H_2_O_2_ sensing in a Parkinson’s disease in vitro model. Sensor. Actuator. B Chem..

[104] Zhu J., Wang L., Xu S., Peng L., Gao Z., Liu S., Xi S., Ma S., Cai W. (2024). A high-performance wearable microneedle sensor based on a prussian blue-carbon nanotube composite electrode for the detection of hydrogen peroxide and glucose. Sensor. Actuator. B Chem..

[105] Li R., Liu X., Qiu W., Zhang M. (2016). In Vivo Monitoring of H_2_O_2_ with Polydopamine and Prussian Blue-coated Microelectrode. Anal. Chem..

[106] Felix F.S., Angnes L. (2018). Electrochemical immunosensors – A powerful tool for analytical applications. Biosens. Bioelectron..

[107] Kongkaew S., Cotchim S., Kanatharana P., Thavarungkul P., Limbut W. (2023). Disposable label-free electrochemical immunosensor based on prussian blue nanocubes for four breast cancer tumor markers. Talanta.

[108] Son S.E., Gupta P.K., Hur W., Choi H., Lee H.B., Park Y., Seong G.H. (2020). Determination of glycated albumin using a Prussian blue nanozyme-based boronate affinity sandwich assay. Anal. Chim. Acta.

[109] Shen C., Wang L., Zhang H., Liu S., Jiang J. (2020). An Electrochemical Sandwich Immunosensor Based on Signal Amplification Technique for the Determination of Alpha-Fetoprotein. Front. Chem..

[110] Zhang Q., Li L., Qiao Z., Lei C., Fu Y., Xie Q., Yao S., Li Y., Ying Y. (2017). Electrochemical Conversion of Fe_3_O_4_ Magnetic Nanoparticles to Electroactive Prussian Blue Analogues for Self-Sacrificial Label Biosensing of Avian Influenza Virus H5N1. Anal. Chem..

[111] Dashtban M., Maki M., Leung K.T., Mao C., Qin W. (2010). Cellulase activities in biomass conversion: measurement methods and comparison. Crit. Rev. Biotechnol..

[112] Chang H., Gacias Amengual N., Botz A., Schwaiger L., Kracher D., Scheiblbrandner S., Csarman F., Ludwig R. (2022). Investigating lytic polysaccharide monooxygenase-assisted wood cell wall degradation with microsensors. Nat. Commun..

[113] Kerchev P., De Smet B., Waszczak C., Messens J., Van Breusegem F. (2015). Redox Strategies for Crop Improvement. Antioxid. Redox Signal..

[114] Dvořák P., Krasylenko Y., Zeiner A., Šamaj J., Takáč T. (2021). Signaling Toward Reactive Oxygen Species-Scavenging Enzymes in Plants. Front. Plant Sci..

